# Reliability and Validity of the Streamlined Wolf Motor Function Test for Chronic Stroke

**DOI:** 10.21315/mjms-09-2024-736

**Published:** 2025-02-28

**Authors:** Su Sandi Hla Tun, Sawitri Wanpen, Nomjit Nualnetr, Uraiwan Chatchawan, Rungthip Puntumetakul, Myitzu Khin

**Affiliations:** 1 School of Physical Therapy, Faculty of Associated Medical Sciences, Khon Kaen University, Khon Kaen, Thailand; 2 Research Center in Back, Neck, Other Joint Pain and Human Performance, School of Physical Therapy, Faculty of Associated Medical Sciences, Khon Kaen University, Khon Kaen, Thailand

**Keywords:** upper extremity, chronic stoke, outcome, motor ability

## Abstract

**Background:**

The present study evaluated the reliability and validity of the Streamlined Wolf Motor Function Test for Chronic Stroke (SWMFT-C), a shortened and redesigned version of the Wolf Motor Function Test (WMFT) to determine upper extremity (UE) motor abilities.

**Methods:**

Twenty individuals with chronic stroke were included in a cross-sectional study design. The Fugl-Meyer Assessment of the Upper Extremity (FMA-UE) and the Stroke Impact Scale (SIS) were used to assess impaired motor recovery of the UE in these patients. The SWMFT-C’s test-retest (two weeks) reliability and inter-rater reliability (three physical therapists) were examined using the intra-class correlation coefficients (ICCs) ICC_2,1_ and ICC_3,1_. Validity was analysed by FMA-UE and SIS–hand function at baseline and 2 weeks using Pearson’s *r* values.

**Results:**

The SWMFT-C performance time(s) demonstrated excellent test-retest reliability (ICC_3,1_ = 0.943, 95% confidence interval [CI] = 0.859–0.978, standard error of measurement [SEM] = 0.15) and outstanding inter-rater reliability (ICC_2,1_ = 0.999, 95% CI = 0.998–1.000, SEM = 1.15). The functional ability scale (FAS) also demonstrated excellent test-retest reliability (ICC_3,1_ = 0.945, 95% CI = 0.861–0.978, SEM = 0.12) and inter-rater reliability (ICC_2,1_ = 0.973, 95% CI = 0.944–0.989, SEM = 0.18). Internal consistency (IC) was calculated using the overall Cronbach’s alpha and demonstrated outstanding agreement as shown by values of 0.99 and 0.94 in performance time(s) and FAS, respectively; the values of minimum detectable change (MDC_95_) were 2.26 seconds and 0.34 seconds, respectively. The validity was good to excellent as correlated with FMA-UE and SIS–hand function, ranging from −0.86 to −0.52 in performance time(s) and 0.65 to 0.80 in FAS.

**Conclusion:**

The SWMFT-C is a valid, reliable clinical instrument for the population with chronic stroke.

## Introduction

Stroke, a major consequence of cerebrovascular disease, is the second most common cause of mortality worldwide (1). Upper extremity (UE) problems after a stroke cause many patients to live with persistent long-term impairments (2), which affect the use of the affected extremities in everyday activities (3). Sensorimotor impairments are common after a stroke, such as wrist pain, muscle weakness, abnormal muscle tone, spasticity, hemiplegic shoulder pain and subluxation, and reduced motor coordination, grip strength, and dexterity (4).

Motor deficits related to a stroke can cause difficulties in the performance of activities of daily living (ADL), reduced participation, and a lower quality of life (5). Grube and colleagues state that outcome measures in the motor domain are essential to monitoring and optimising realistic treatment goals and to ensure transparency regarding the quality of care during stroke rehabilitation (6).

In stroke rehabilitation, the use of evidence-based standardised outcomes is important in assessing motor ability, supporting individualised rehabilitative planning, and evaluating the patient’s improvement. Moreover, the effectiveness of rehabilitation programmes can be evaluated to support healthcare professionals through the development of new knowledge (7). Healthcare professionals increasingly emphasise the achievement of successful rehabilitation and of the expected outcomes of targeted changes in the stroke recovery process.

The measurement of UE function is critical for improving clinical practice, for research purposes, and for evaluating the efficacy of rehabilitation interventions. Many valid and reliable outcome measures exist for the UE, some of which are integrated to provide a more comprehensive view of functioning (8). Among the assessment tools for the motor ability of the UE, the Wolf Motor Function Test (WMFT) is one of the more widely used performance measures; it examines performance time(s) and functional ability through two strength tasks and 15 functional tasks that range from basic to complex and from proximal to distal (9). The WMFT is recognised as one of the gold standard outcome measures in evaluating motor recovery. It demonstrates high reliability and sensitivity in assessing time-based outcomes for stroke patients across various levels of severity and different stages of recovery (10).

During the administration of the WMFT, patients are required to complete 17 measurement items. The complexity and length of the instructions have led to patient fatigue and confusion, especially in those who were severely impaired, which in turn affects their performance (9). This has resulted in incomplete tasks, misleading outcomes, inaccurate scores, and undesirable results (10, 11). Streamlining the WMFT is essential, as doing so can reduce administration time and give the clinician the best possible information regarding the patient’s chances of recovery, as the tasks are intended to highlight the movements of specific joints (14, 15). The Streamlined WMFT for Chronic Stroke (SWMFT-C) is an easily administered, unidimensional measuring scale that is properly focused for UE motor function assessment in chronic stroke survivors (14). The UE motor ability difficulty test items measure the patient’s functional ability over a range of UE motor functions and provide information on their progress toward functional status recovery (14). This six-item variant of the WMFT has exhibited enhanced clinical value focused on patients with chronic stroke when compared to the original WMFT scale. The six points on the functional ability scale (FAS) range from 0, “no use” to 5, “normal” (15). The performance time(s) is similar to that of the WMFT, and the time for each item is typically truncated to 120 seconds (13, 15). The SWMFT-C exhibits no significant difference in sensitivity from the original WMFT performance time(s) (13).

The SWMFT-FAS has demonstrated excellent reliability in patients with chronic stroke and shown good predictive validity and fair criterion validity in patients with subacute stroke (13, 14, 16). In addition, the SWMFT log performance time(s) show the internal responsiveness of effect size and standardised response mean in chronic stroke (13, 16). Nevertheless, limited research has considered SWMFT-C performance time(s) and FAS in their application to UE motor ability in patients with chronic stroke. Therefore, a standardised outcome with excellent inter- and intra-reliabilities, internal consistency (IC), minimum detectable change (MDC_95_), and validity needed to be tested. Consequently, the present research aimed to determine the reliability and validity of the SWMFT-C in patients who had experienced chronic stroke.

When evaluating UE motor function, excellent clinimetric properties, such as reliability, validity, and responsiveness, are crucial for prognostic and diagnostic purposes (17), so it is vital that these clinimetric properties be thoroughly demonstrated in clinical trials to support using the outcome measures (18). Reliability indicates the degree to which the measurement is influenced by measurement errors or the extent to which the measurement is inherently reproducible (19). Construct validity describes the ability of a measure to assess an abstract concept or construct (20). The present study used convergent validity to determine the correlation between the variables that relate to the same construct. The researchers assessed the UE motor ability of patients with chronic stroke using the SWMFT-C over a two-week interval as in a previous study (21). No treatment was given to them during the two weeks.

The SWMFT-C is a standardised, non-invasive tool to assess motor ability in the UE and is useful in clinical trials due to its excellent reliability in FAS (14) and good validity and responsiveness in performance time(s) (13, 16). The WMFT shows a high correlation with the Fugl-Meyer Assessment of the Upper Extremity (FMA-UE) in stroke patients with mild and moderate impairments (11, 22). Furthermore, a previous study reports moderate associations between the SWMFT-FAS and the shortened FMA-UE and the Stroke Impact Scale (SIS) in subacute stroke patients (16). Another study observed correlations between log-transformed SWMFT performance time(s) and FMA-UE in subacute stroke patients (13). However, the reliability (including inter- and intra-rater reliabilities, IC, and MDC_95_) and validity of both scales need to be evaluated in patients with chronic stroke. Consequently, this study investigated the reliability and validity of the SWMFT-C in patients with chronic stroke over a two-week interval.

## Methods

### Study Design

This cross-sectional study on patients with chronic stroke was carried out from October 2022 through March 2023 at the Physical Medicine and Rehabilitation Department, North Okkalapa General Hospital, Yangon, Myanmar. Prior to starting the study, each participant signed a written informed consent statement before any data were collected.

### Participants

Demographic data and stroke history were collected from 20 patients with chronic stroke (referred by physicians) who were screened for eligibility. The inclusion criteria embraced patients aged 40–79 years old with both ischaemic and haemorrhagic stroke; hemiplegia induced by a stroke that occurred at least 6 months from initial onset; ability to sit for at least 30 minutes; a motor portion of FMA-UE score between 19 and 58; the ability to initiate active extension of the wrist and fingers; right-handedness as determined by the Edinburgh Handedness Inventory; a Mini Mental State Examination score above 24; and willingness to participate in the study. Patients were excluded who had other neurological diseases (such as Parkinson’s disease, dementia, Alzheimer’s disease, peripheral neuropathy, or bilateral stroke), musculoskeletal problems (such as deformities, recent fracture, or reflex sympathetic dystrophy in the affected UE), pain that interfered with the functional use of the UE as identified by a pain/ROM FMA-UE pain score of 1.0 for at least two joints on the paretic arm as well as those who had been in a serious accident, had any disease limiting the function of the UE before the stroke, exhibited shoulder pathology, had undergone recent surgery to the neck, arm, or shoulder, or had treatment requiring any surgical incision.

### Procedures

#### Training Raters to Use the SWMFT-C

The assessment procedure for the SWMFT-C was performed according to the WMFT manual (23). The study’s three raters were physical therapists; one was an experienced expert in administering and scoring the SWMFT-C, whereas the other two physical therapists were novice raters, including the principal investigator and another therapist. The expert rater had extensive experience of using the SWMFT-C as an outcome measure in doctoral research as well as 30 years of experience in the rehabilitation of stroke patients. Additionally, both novice raters had 10 years of experience in the rehabilitation of stroke patients. Prior to assessing the patient’s performance, the expert physical therapist trained the two novice raters on accurately assessing and scoring the SWMFT-C. All the raters were provided with a scoring manual and directions for administering the SWMFT-C. Subsequently, the three raters watched and individually scored each of the video recordings to ensure inter-rater reliability. The two novice raters were trained to 100% agreement for the FAS and within 0.5 seconds for performance time(s).

#### Test Administration

The principal investigator explained and demonstrated each item of the SWMFT-C to the patients. Each patient first received instructions from the principal investigator on how to complete the task, and the investigator then demonstrated the process to the patient, with each task explained and demonstrated twice by the principal investigator. The patients refrained from practicing the task during the principal investigator’s description and demonstration. It is important that patients complete all the assigned tasks as quickly as possible, so they attempted to complete each task as fast as possible to a maximum of 120 seconds. Every patient’s performance was recorded on camera to ensure a precise evaluation of all performance standards. Prior to the SWMFT-C, the principal investigator assessed the patients in the FMA-UE and SIS–hand function.

The patients performed the entire SWMFT-C for a second time two weeks after the baseline assessment. The patients did not receive any treatment and were advised not to practice any of the tasks from the SWMFT-C during the two-week interval. The performance of each patient in the second performance was assessed by the principal investigator and recorded on camera. The three raters separately reviewed the video recordings of the patients’ performance to determine inter-rater reliability.

### Sample Size

The sample size was evaluated using ICC in STATA version 10. Three repeated measurements calculated an ICC of 0.91 for the hypothesis value (*p*_1_) (14) in the SWMFT-C and an ICC of 0.78 for the null value (*p*_0_); the power was 80%. The evaluation indicated that 20 patients with chronic stroke were required.

### Material and Assessment Tools

#### Streamlined WMFT for Chronic Stroke

The SWMFT-C, a shortened form of the WMFT, comprises six tasks: extending the elbow 28 cm on a tabletop against a 0.4536-kg (1-lb) weight; moving hand to box (front); lifting a can; lifting a pencil; turning a key in a lock; and folding a towel (14). The FAS is scored on a six-point scale ranging from 0 (no use) to 5 (normal) (15). Before proceeding to the next test item, the patients were advised to perform each of the tested movements twice with their affected extremity after completing the movement with their non-affected extremity (12). For every test item, the mean of the two measurements was computed for the median and mean rate values (24). For the performance time scale, the six items should be performed as quickly as possible; the time allowed for each item was typically truncated to 120 seconds (13).

#### Fugl-Meyer Assessment Upper Extremity

The FMA is a performance-based measure (25). To assess a particular construct, it is divided into five subsections: motor, balance, sensory, range of motion, and pain. The motor component of the FMA assesses coordination, reflexes, speed, and movement. Fugl-Meyer et al. divide the motor portion into two subsections: UE (upper arm, wrist, and hand) and lower extremities (25). The present study employed the UE section because the FMA-UE is regarded as a gold standard outcome measure (26) and is reliable and well validated (27). From a total possible score of 66, a sub-score of 36 was assigned to the upper arm (FMA-UA) and a sub-score of 30 to the wrist and hand (FMA-WH). A three-point ordinal scale was used to assess the majority of the items, with 0 representing no function, 1 partial function, and 2 complete function (28).

#### Stroke Impact Scale

The 59 items of the SIS version 3.0 (29) measure eight domains: mobility, emotion, memory/thinking, participation, hand function, ADL/instrumental (IADL), strength, and communication. The scale has been shown to be valid and reliable (30–32). This study assessed the SIS–hand function domain.

### Data Analysis

The participants’ demographic data and clinical characteristics were analysed using IBM Statistical Package for the Social Sciences (SPSS) version 28. The reliability of the SWMFT-C was analysed for continuous data (total scores).

The IC of each SWMFT-C item’s ordinal scale was assessed using measurements taken by the first rater (an expert). The Cronbach’s alpha coefficient and adjusted item-total correlation calculations were part of this process. A Cronbach’s alpha of > 0.7 was deemed acceptable (33), and an item-total correlation value of > 0.2 was considered equivalent (34). The single-item agreement of the SWMFT-C score between the three raters and between two assessments (third rater) was calculated using the weighted kappa coefficient (33). The Shapiro-Wilks test was used to assess the data measurements for the continuous SWMFT-C score data before reliability was analysed.

The intra-class correlation coefficient (ICC) was used to separately examine the inter-rater reliability using model 2, 1 (two-way random effects, or ICC_2,1_) and the intra-rater reliability using model 3, 1 (two-way mixed effects, or ICC_3,1_). The inter-rater reliability of the six SWMFT-C tasks scored by the first rater (an expert) and the second and third rater was investigated. To investigate the intra-rater reliability, the data for the six SWMFT-C tasks assessed by the third rater on the first and second occasions were used.

The standard error of measurement (SEM) was computed using the ICC results for test-retest and inter-rater reliability, and the MDC_95_ was determined by multiplying the SEM × *z* value × √2 (37). The third rater’s data were used to compute the MDC.

Construct validity was calculated by correlating the average scores of SWMFT-C performance time(s) and FAS and those of FMA-UE and SIS–hand function by using Pearson correlation (*r*) at baseline and at two weeks. The FMA-UE and SIS–hand function were selected to correlate because of their similar constructions to those of the SWMFT-C (11). A correlation of > 0.75 is considered excellent, 0.50–0.75 good, and 0.25–0.50 fair (20).

## Results

Table 1 shows the patients’ demographic characteristics. The overall Cronbach’s alpha of statistics for all items is 0.99 in performance time(s) and 0.94 in FAS, confirming excellent agreement. In performance time(s), almost all the items show satisfactorily high correlations ranging from 0.71 to 0.89 except item 1 (extend elbow) and item 6 (fold towel), as the two items show moderate correlation. The weighted kappa values for each item reveal substantial to almost perfect single-item agreement. The weighted kappa values between raters range from a low of 0.72 for item 4 (lift pencil) to a high of 0.85 for item 6 (fold towel). The weighted kappa values for the test-retest agreement coefficient range from 0.45 for item 2 (hand to box [front]) to 0.75 for item 1 (extend elbow) as shown in Table 2. For FAS, almost all the items returned satisfactory moderate correlations ranging from 0.41 to 0.68 except item 4 (lift pencil) and item 6 (fold towel), with the two items showing high correlation. The weighted kappa values for each item indicate substantial to almost perfect single-item agreement except for item 6 (fold towel). The weighted kappa value between raters ranges from a low of 0.36 for item 6 (fold towel) to a high of 0.91 for item 2 (hand to box [front]). The weighted kappa values for the test-retest agreement coefficient range from 0.21 for item 6 (fold towel) to 0.90 for item 2 (hand to box [front]).

Table 3 presents the IC of the SWMFT-C performance time(s), test-retest reliability, and inter-rater reliability. High reliability is shown by an ICC of 0.943 (95% CI = 0.859 to 0.978, *p <* 0.001, SEM = 0.15) for test-retest reliability and an ICC of 0.999 (95% CI = 0.998 to 1.000, *p <* 0.001, SEM = 1.15) for inter-rater reliability. The inter-rater reliabilities between rater 1 and 2, rater 2 and 3, and rater 1 and 3 are highly significant (ICC = 0.998 to 0.999, *p <* 0.001). The MDC_95_ and IC are 2.26 and 0.793, respectively.

Table 4 shows the inter-rater and test-retest reliability and IC of SWMFT-C-FAS. The inter-rater reliability proved highly reliable, with an ICC of 0.973 (95% CI = 0.944 to 0.989, *p <* 0.001, SEM = 0.18). Similarly, the test-retest reliability was found to be highly reliable, with an ICC of 0.945 (95% CI = 0.861 to 0.978, *p <* 0.001, SEM = 0.12). The inter-rater reliabilities between rater 1 and 2, rater 2 and 3, and rater 1 and 3 are highly significant (ICC = 0.943 to 0.999, *p <* 0.001). The MDC_95_ and IC are 0.34 and 0.905, respectively.

Table 5 provides the construct validity of the SWMFT-C by the correlation method (Pearson’s *r*) with FMA-UE and SIS–hand function at baseline and at 2 weeks. At baseline assessment, the performance time(s) show significantly negative correlations with FMA-UE (*r* = −0.71, 95% CI = −0.88 to −0.38, *p <* 0.001) and SIS–hand function (*r* = −0.86, 95% CI = −0.94 to −0.66, *p <* 0.001). The FAS shows significantly positive correlations with FMA-UE (*r* = 0.80, 95% CI = 0.54 to 0.91, *p <* 0.001) and SIS–hand function (*r* = 0.69, 95% CI = 0.34 to 0.86, *p <* 0.001). At two weeks, the performance time(s) show significantly negative correlations with FMA-UE (*r* = −0.52, 95% CI = −0.78 to −0.10, *p* = 0.020) and SIS–hand function (*r* = −0.74, 95% CI = −0.89 to −0.43, *p <* 0.001). The FAS shows significantly positive correlations with the FMA-UE (*r* = 0.71, 95% CI = 0.39 to 0.88, *p <* 0.001) and SIS–hand function (*r* = 0.65, 95% CI = 0.28 to 0.84, *p* = 0.002).

## Discussion

The present study investigated the reliability and validity of SWMFT-C in patients with chronic stroke and found that the SWMFT-C demonstrates reliable psychometric properties and high-quality outcome measurements for UE motor ability assessment in patients with chronic stroke. The investigation’s outcomes support our hypothesis. The SWMFT-C shows excellent reliability in both performance time(s) and FAS as well as excellent correlation with the FMA-UE and SIS–hand function in the current study. The ICs returned satisfactory values in performance time(s) and FAS, with Cronbach’s values of 0.99 and 0.94, respectively. The item to total correlation was determined to have the lowest value for item 1 (extend elbow) in performance time(s) and item 2 (hand to box) in FAS.

The correlation between the ratings given by the three raters when observing videos was assessed by inter-rater reliability. The high inter-rater reliabilities in the current study are in line with the previous study conducted in FAS only, which returned a reliability coefficient of 0.9 (14). The test-retest reliabilities of both performance time(s) and FAS scores are also high. In addition, the SEM is a better measure than reliability for assessing the quality of an outcome, and a smaller SEM had higher accuracy in conducting the outcome (36). In the current study, both the SWMFT-C scores yielded small SEM values, indicating that SWMFT-C offers an almost perfectly reliable outcome in the assessment of patients with chronic stroke. Furthermore, an obvious change in motor ability is indicated by the MDC_95_ values, which show lower detectable change scores. These results are the first results for both scores of this outcome. The construct validity of the performance time(s) is negatively correlated with the FMA-UE and SIS–hand function. Also, FAS is positively correlated with the FMA-UE and SIS–hand function.

Bogard and colleagues (2009) suggested that further study was needed to validate the SWMFT-C against another standard test, such as the FMA-UE (12). The present study found a high correlation between the FMA-UE and the SWMFT-C; both scores show that the FMA-UE has an excellent negative correlation with the performance time(s) of the SWMFT-C; the more impaired the UE functions, the longer it took to accomplish the activities. Moreover, the FMA-UE has an excellent positive correlation with the SWMFT-C-FAS; the less impaired the UE functions, the better the performance in functional activities. The findings of the current study confirm the excellent construct validity (or criterion validity) of the SWMFT-C. Because of the strong correlation coefficient between the FMA-UE and the SWMFT-C in analysing external responsiveness, assessing one outcome can predict the result of an untested one, and changes in one outcome can predict changes in another in clinical settings (17). Similarly, the study of Fu et al. found significantly strong associations between the SWMFT-FAS and the shortened FMA (*r* = 0.57 to 0.68, *p* < 0.001) and SIS (*r* = 0.39 to 0.58, *p* < 0.001) in patients with subacute stroke (14). Another similar study reports significantly good concurrent validity (ρ = 0.69, *p* < 0.01) and predictive validity (ρ = 0.68, *p* < 0.01) between the FMA-UE and the SWMFT-C log performance time(s) only in patients with subacute stroke (13). The present study fills the gap in the previous research that is the correlation of standard outcome, the original FMA-UE and the SWMFT-C, both in performance time(s) and FAS in patients with chronic stroke. Therefore, the SWMFT-C can be useful for selecting an outcome in clinical trials.

Most outcome measures used in stroke rehabilitation are based on the severity of impairment. In contrast, the SWMFT-C, derived from the original WMFT, was developed based on the chronicity of patients, resulting in two versions: one for subacute stroke and one for chronic stroke (37). The SWMFT-C is designed to assess UE motor functions specifically in chronic stroke patients. Its compact format of six items minimises the time and energy expended on unnecessary tasks, allowing patients to complete other outcome measures and/or perform additional rehabilitation programmes. Consequently, the SWMFT-C is regarded as a highly sensitive and optimally well-suited tool for assessing the target population (37, 38). Due to its significant psychometric properties, healthcare professionals specialising in neurorehabilitation can widely apply the SWMFT-C to implement and adjust treatment programmes.

Sociocultural factors can influence rehabilitation by affecting both patient support and the use of technologies that either facilitate or impede the quality of treatment (39). However, in the administration of the SWMFT-C, the materials used are derived from locally accessible and affordable resources. The test items are designed as meaningful tasks that are not overly complicated for assessors to evaluate. Consequently, the implementation of the SWMFT-C addresses sociocultural challenges by meeting the functional requirements of chronic stroke patients in diverse geographical settings.

This study has limitations. First, only patients with chronic stroke were assessed in this study. Second, the patients had no cognitive impairment. Third, the patients had moderate deficits in their UE functions. More studies are warranted to assess patients with different types of stroke, different cognitive abilities, and different levels of motor deficit to enhance the generalisability of the results.

## Conclusion

More clinical trials are being conducted for stroke rehabilitation; therefore, it is critical to determine which assessment tools are best for evaluating the results of UE motor function interventions. The SWMFT-C demonstrated high reliability in assessing the UE motor ability of patients with chronic stroke, consistent with the previous study. Furthermore, the SWMFT-C shows a good to excellent correlation with the FMA-UE and SIS–hand function. The clinical implications of this standardised tool should be communicated to healthcare professionals in the rehabilitation field. In stroke rehabilitation centres, hospitals, outpatient departments, and private practices, clinicians should receive ongoing training programmes for treatment and assessment, including the SWMFT-C. This will enable accurate assessment of chronic stroke patients using the SWMFT-C, facilitating the recording of data for both rehabilitation and research purposes and promoting its extensive use in rehabilitation.

## Figures and Tables

**Table 1:** Demographic characteristics of patients (*n* = 20)

Demographic characteristics	*n* (%)
Age (years)^a^	59.55 (7.62)

Gender	
Male	18 (90)
Female	2 (10)

Education	
Primary school	2 (10)
Secondary school	16 (80)
High school	2 (10)

Occupation	
Non-working	16 (80)
Working	4 (20)

Smoking and alcohol drinking	
Former smoker	5 (25)
Former alcohol drinker	15 (75)

Post-stroke duration (months)^a^	50.25 (29.67)

Side of stroke	
Right	10 (50)
Left	10 (50)

Dominant hand	
Right	20 (100)
Left	0 (0)

Type of stroke	
Ischaemic	14 (70)
Haemorrhagic	6 (30)

Number of stroke(s)	
One	18 (90)
More than one	2 (10)

Comorbidities	
Hypertension	9 (45)
IHD	11 (55)

Having somatosensory loss in UE	
Not having	15 (75)
Having	5 (25)

MMSE^a^	26.05 (0.95)

FMA-UE^a^	32.40 (7.83)

Notes:

a mean (SD);

FMA-UE = Fugl-Meyer Assessment Upper Extremity; IHD = ischaemic heart disease; MMSE = Mini Mental State Examination; SD = standard deviation

**Table 2:** Inter-rater and test-retest agreement for a single-item of the SWMFT-C and item-total correlation from *n* = 20

Item	Performance time(s)			Functional ability scale		

	Single-item agreement (weighted kappa)		Item-total	Single-item agreement (weighted kappa)		Item-total
	
	Inter-rater	Test-retest		Inter-rater	Test-retest	
Extend elbow	0.78	0.75	0.55	0.64	0.78	0.49
Hand to box (front)	0.73	0.45	0.83	0.91	0.90	0.41
Lift can	0.81	0.64	0.83	0.62	0.65	0.54
Lift pencil	0.72	0.55	0.71	0.69	0.57	0.83
Turn key	0.78	0.67	0.89	0.63	0.68	0.68
Fold towel	0.85	0.67	0.70	0.36	0.21	0.80

Note: SWMFT-C = Streamlined Wolf Motor Function Test for Chronic Stroke

**Table 3:** Mean (SD), inter-rater and test-retest reliability, and internal consistency of SWMFT-C performance time(s) (*n* = 20)

	Rater	Mean (SD)	Inter-rater reliability ICC_2,1_ (95% CI) *P*-value^a^	Test-retest reliability ICC_3,1_ (95% CI) *P*-value^a^
First assessment	1	5.18 (3.13)		
	2	5.13 (3.12)	0.999 (0.998, 1.000)	0.943 (0.859, 0.978)
	3	5.17 (3.09)	*P <* 0.001*	*P <* 0.001*
Second assessment	3	4.92 (3.74)		

MDC_95_		2.26		

Internal consistency (alpha)		0.793		

Notes:

a two-way random, single measures, absolute agreement;

b two-way mixed, single measures, consistency;

ICC = intra-class correlation coefficient; ICC_2,1_ = two-way random effects; ICC_3,1_ = two-way mixed effects; CI = confidence interval; SD = standard deviation; MDC = minimum detectable change; SWMFT-C = Streamlined Wolf Motor Function Test for Chronic Stroke;

* MDC was calculated from the data of two assessments of the third rater

**Table 4:** Mean (SD), inter-rater and test-retest reliability, and internal consistency of SWMFT-C functional ability scales (*n* = 20)

	Rater	Mean (SD)	Inter-rater reliability ICC_2,1_ (95% CI) *P*-value^a^	Test-retest reliability ICC_3,1_ (95% CI) *P*-value^b^
First assessment	1	2.98 (0.55)		
	2	3.05 (0.67)	0.973 (0.944, 0.989)	0.945 (0.861, 0.978)
	3	2.99 (0.55)	*P <* 0.001*	*P <* 0.001*
Second assessment	3	2.98 (0.51)		

MDC_95_		0.34		

Internal consistency (alpha)		0.905		

Notes:

a two-way random, single measures, absolute agreement;

b two-way mixed, single measures, consistency;

ICC = intra-class correlation coefficient; ICC_2,1_ = two-way random effects; ICC_3,1_ = two-way mixed effects; CI = confidence interval; SD = standard deviation; MDC = minimum detectable change; SWMFT-C = Streamlined Wolf Motor Function Test for Chronic Stroke;

* MDC was calculated from the data of two assessments of the third rater

**Table 5:** Construct validity between SWMFT-C, FMA-UE, and SIS–hand

Criterion measure	Baseline assessment scores			

	SWMFT-C performance time(s)		SWMFT-C-FAS	

	*r* (95% CI)	*P*-value^a^	*r* (95% CI)	*P*-value^a^
FMA-UE	−0.71** (−0.88, −0.38)	< 0.001	0.80** (0.54, 0.91)	< 0.001
SIS–hand function	−0.86** (−0.94, −0.66)	< 0.001	0.69** (0.34, 0.86)	< 0.001

**Criterion measure**	**Two-week assessment scores**			

	**SWMFT-C performance time(s)**		**SWMFT-C-FAS**	

	***r*** ** (95% CI)**	***P*** **-value** a	**r (95% CI)**	***P*** **-value** a

FMA-UE	−0.52* (−0.78, −0.10)	0.020	0.71** (0.39, 0.88)	< 0.001
SIS–hand function	−0.74** (−0.89, −0.43)	< 0.001	0.65* (0.28, 0.84)	0.002

Notes:

a Pearson’s correlation;

CI = confidence interval; FMA-UE = Fugl-Meyer Assessment Upper Extremity; SWMFT-C = Streamlined Wolf Motor Function Test for Chronic Stroke; FAS = functional ability scale; *r* = Pearson’s correlation coefficient
